# Does Economic Growth Reduce Childhood Undernutrition in Ethiopia?

**DOI:** 10.1371/journal.pone.0160050

**Published:** 2016-08-10

**Authors:** Sibhatu Biadgilign, Arega Shumetie, Habtamu Yesigat

**Affiliations:** 1 Public Health Nutrition Research, Addis Ababa, Ethiopia; 2 Department of Economics, Haramaya University of Ethiopia and Makerere University of Uganda, Harar, Ethiopia; 3 Production and Resource Economics, Technical University Munich, Freising, Germany; Western Sydney University, AUSTRALIA

## Abstract

**Background:**

Policy discussions and debates in the last couple of decades emphasized efficiency of development policies for translating economic growth to development. One of the key aspects in this regard in the developing world is achieving improved nutrition through economic development. Nonetheless, there is a dearth of literature that empirically verifies the association between economic growth and reduction of childhood undernutrition in low- and middle-income countries. Thus, the aim of the study is to assess the interplay between economic growth and reduction of childhood undernutrition in Ethiopia.

**Methods:**

The study used pooled data of three rounds (2000, 2005 and 2010) from the Demographic and Health Surveys (DHS) of Ethiopia. A multilevel mixed logistic regression model with robust standard errors was utilized in order to account for the hierarchical nature of the data. The dependent variables were stunting, underweight, and wasting in children in the household. The main independent variable was real per capita income (PCI) that was adjusted for purchasing power parity. This information was obtained from World Bank.

**Results:**

A total of 32,610 children were included in the pooled analysis. Overall, 11,296 (46.7%) [46.0%-47.3%], 8,197(33.8%) [33.2%-34.4%] and 3,175(13.1%) [12.7%-13.5%] were stunted, underweight, and wasted, respectively. We found a strong correlation between prevalence of early childhood undernutrition outcomes and real per capita income (PCI). The proportions of stunting (r = -0.1207, p<0.0001), wasting (r = -0.0338, p<0.0001) and underweight (r = -0.1035, p<0.0001) from the total children in the household were negatively correlated with the PCI. In the final model adjustment with all the covariates, economic growth substantially reduced stunting [β = -0.0016, SE = 0.00013, p<0.0001], underweight [β = -0.0014, SE = 0.0002, p<0.0001] and wasting [β = -0.0008, SE = 0.0002, p<0.0001] in Ethiopia over a decade.

**Conclusion:**

Economic growth reduces child undernutrition in Ethiopia. This verifies the fact that the economic growth of the country accompanied with socio-economic development and improvement of the livelihood of the poor. Direct nutrition specific and nutrition sensitive interventions could also be recommended in order to have an impact on the massive reduction of childhood undernutrition in the country.

## Introduction

Policy discussions and debates in the last couple of decades emphasized on the efficiency of development policies for translating economic growth to development [1–4]. There are a number of reasons for this. First, improved human capital and nutritional status coupled with economic growth are basic elements of better societal wellbeing. Second, improved nutrition enhances the physical and mental working capacity, productivity and earnings, which in turn contributes to economic and social development [1–3, 5]. In addition to the moral and ethical grounds that promote societal health, these all support the role of improved nutrition and societal health for productivity, growth and transformation of a given country.

Some critical interventions have been underway in sub-Saharan Africa and the rest of developing world that particularly target towards reducing the level of undernutrtion. For instance, targeted and pro-poor economic strategies or agricultural transformation strategies can contribute to reduce rural poverty and inequality [2, 4, 6, 7]. Nonetheless, rapid economic growth brought mixed results in terms of human development in many countries of the developing world and remains inconclusive [2, 8–10]. In some countries, rapid economic growth accompanied with increasing income inequality, and others show little or no substantial improvement in poverty and nutrition outcomes [2, 8, 9, 11]. Countries that have made a good progress for changing the nutrition and health of their population applied nutrition-specific interventions coupled with economic growth and social sector development [12].

For the past decade, Ethiopia has achieved economic progress; and registered a gross domestic product (GDP) growth rate of 11 percent per annum [13]. Despite this progress, the level of micronutrient deficiencies still high and the feeding practices of Ethiopian families remain sub-optimal [13]. The country has been in a transition in nutrition and other relevant interventions, and they often can play a crucial role for the reduction of nutritional situations [14].

Financial incentives and cash transfer are often used to alleviate poverty and reduce structural barriers [12]. They argue that improved income could further be translated to improved nutrition and child care. Other studies indicate that improvement in nutritional status doesn't coincide with income growth, even in the short run [15]. This may require specific investments in human resources and other interventions [1, 3, 4, 15]. In the same line, a study done in India stated that, economic growth didn’t bring a significant change in the level of risk of underweight, stunting and wasting. This paper suggested the direct investments in appropriate health interventions as a necessary conditions to reduce childhood undernutrition [16]. Recent findings in other low and middle-income countries(LMIC) depict a very small to null association between increment in per capita income and reductions in early childhood undernutrition [17]. Those studies emphasize on the need for direct investments to improve nutritional status of children [1, 3, 4, 17]. In contrast from this, sustainable economic growth can bring a significant reduction in malnutrition [18]. Some studies also indicated that income growth can contribute to poverty reduction and improved nutritional status. They argue that improved income for the poor can significantly improve their food expenditures, enhance their access to health care services, water and sanitation [1, 2, 11]. In Vietnam, economic growth contributed to substantial improvement in child height in the 1990s [19]. Similarly, an increase in GDP per capita between 1970 and 1995 contributed to total reduction in the prevalence of child undernutrition [20].

In general, there are mixed evidences on the interplay between economic growth, and child nutritional and health status. The impact of economic development on nutrition improvement could be substantial in sub-Saharan Africa where a significant proportion of the population is poor. With this regard, a failure to invest in child health and nutrition can lead to severe long-term economic consequences both at the household and macro-economic level [21]. Within this stand, there is a dearth of literature to refute the hypothesis on the association between economic growth and reduction in childhood undernutrition in low and middle income countries. Thus, this study aimed is to assess the interplay between economic growth and childhood undernutrition in Ethiopia.

## Methods

### Study setting and context

We used a dataset of Ethiopian Demographic and Health Survey (DHS) from three rounds (2000, 2005, and 2010). The DHS is a collaborative project between the Central Statistical Authority of Ethiopia and ORC Macro of the USA through the MEASURE DHS project. The DHS dataset used here is a repeated cross-sectional survey type that was collected in multiple time points (2000–2010) from different households. The study design was a population based cross sectional study that was conducted in a representative sample of women who were in their reproductive age together with their under five children in Ethiopia. A nationally representative sample of households was considered through a multi-stage cluster sampling technique taking into account for stratification and clustering. The cluster and household allocation by region and residence was a function of the average number of women 15–49 per household.

### Measurements

DHS survey used standardized questionnaires to ask women of reproductive age about their socio-demographic characteristics, maternal and child health, and children nutritional status indicators based on anthropometric measurements. Data were collected by trained enumerators using standardized, well-structured and pre-tested questionnaire. The questionnaire was originally developed in English and then translated into three major local languages- Amharigna, Oromiffa, and Tigrigna. For this study, the data regarding household level, women and child anthropometry were used accordingly. The dependent variable was binary type and, children of below -2 standard deviations of the WHO median reference for height-for-age, weight-for age and weight-for-height are stunted, underweight and wasted respectively. Real per capita income (PCI) measured in constant prices and adjusted for purchasing power parity from World Bank development indictor was used as an independent variable [22]. The other covariates we used for control were socio-economic and demographic status and community level factors (age and sex of the child, age of women, region, place of residence, sex of household head, wealth index quintile, type of toilet facility, source of drinking water, maternal height, respondent's and partner's occupation level, number of household members, partner’s education level and number of under five children in the household) were used for this analysis.

### Data collection procedure

The quantitative data was conducted by interviewing teams which carried out the data collection process. Height and weight measurements were carried out on children under age of five in all selected households. Weight measurements were obtained using lightweight, SECA mother-infant scales with digital screens, designed and manufactured under the guidance of UNICEF. Height measurements were carried out using a measuring board. Children younger than 24 months were measured for height while lying down, and older children, while standing. This study focuses on the individual and household attributes of women and children in Ethiopia. In this study, height and weight measurements of the children were done by taking age and sex into consideration, and converted into Z-scores based on the World Health Organization (WHO) reference population.

### Statistical analysis

Quantitative data of three data sets was pooled and checked for completeness and consistency. Data entry was carried out using SPSS and analysis was done using STATA 12.0 (Stata Corporation, College Station, TX). The Z score values for Length/height-for-age, weight-for-age, weight-for-length, relative to the WHO 2007 reference were constructed. Multilevel mixed logistic regression models with robust standard errors were utilized taking into account hierarchical nature of the data. The regression models were estimated and fitted with wasting, stunting and underweight as binary type dependent variables along with other covariates. At the final model, those variables that retained at P<0.05 were considered to be statistically significant and used to interpret the finding of the study. The method of backward elimination of these variables as potential predictors was used in the final interpretation of the findings. All analyses were done by adjusting for cluster factor (census enumeration areas /selected kebele) and survey-year random effects.

### Model specification

Three models containing exposure variables were fitted into the model building exercise with the outcome of interest. Model 1: considers only PCI as an independent variable, Model 2: includes Model 1 and individual factors (current age and sex of the child) and lastly Model 3: takes into account Model 2 and other covariates like community and household level (age of women, region, place of residence, sex of household head, wealth index quintile, type of toilet facility, source of drinking water, maternal height, respondent’s and partner's occupation level, number of household members, partner’s education level and number of under five children in the household).

### Outcome

The three indices are expressed as standard deviation units from the median of the WHO reference population /reference group determined as stunting (height-for age), underweight (weight-for age) and wasting (weight-for height)- when a child Z-score is below minus two standard deviations (−2 SD) from the median of the reference population. Similarly, this study defined improved sanitation, as a collective sum up of flush toilet; piper sewer system; septic tank; ventilated improved pit latrine (VIP); pit latrine with slab; composting toilet. Improved sources of drinking water include piped water into the dwelling; piped water to yard /plot/compound; public tap or standpipe; tube well or bore well; protected dug well; protected spring [WHO/UNICEF Joint Monitoring Programme (JMP) definition for improved and unimproved sanitation and drinking water sources (WHO/UNICEF 2012)] [23].

### Ethical consideration

Ethical clearance for this study was obtained at a higher level, Ethiopia Health and Nutrition Research Institute (EHNRI) Review Board, the National Research Ethics Review Committee (NRERC) at the Ministry of Science and Technology, the Institutional Review Board of ICF International, and the United States Center for Disease Control and Prevention (CDC) were approved during the initial design and protocol development for the whole survey. For further analysis of the data, prior approval from ORC Macro international was secured from the owner of the raw data. Since we retrieved the already collected data, the participant's records was anonymized and de-identified prior to analysis.

## Results

This study included children aged ranging from 0–59 months from the national representative data from 2000–2010. In these survey rounds, the mothers of the children were interviewed to assess the health, nutritional and other characteristics of the selected children.

A total of 32,610 children were included in the pooled analysis with 11,095(34.02%), 9,861(30.24%) and 11,654(35.74%) samples were considered in 2000, 2005 and 2010 respectively. We integrated the PCI of the country from the World Bank ($515, $612 and $876) in the respective years. The mean age (SD) of the children was 29.18(17.3) months and their mothers’ was 29.2(6.86) years. About 9,589(29.4%) of the respondents were in the age group of 25–29 years. Around 16,653(51.1%) of the children were male. A small proportion (2.26%) of the mothers had a height of less than 145 cm. About 84.4% of them were from the rural part of the country. About 83.9% of the respondents were male headed, 89.9% were married and 24.8% of the respondents were in the lowest wealth quantile during survey times.

Concerning the household environment (drinking water and household sanitation facilities), more than 15.1% of families use unimproved toilet facilities, while 45.6% get improved water. About 53.3% of the respondents didn't engage in paid work while 7,130(22.1%) and 24,673(76.6%) of husbands were employed in paid jobs and agricultural activities respectively [Table 1].

**Table 1:** Distribution of the respondents with background and household characteristics among children age 6–59 months in Ethiopia.

Characteristics	Number (N)	Proportion (%)
Age of child		
0 years	6,137	20.76
1 years	5,503	18.61
2 years	5,731	19.39
3 years	6,231	21.08
4 years	5,961	20.16
Sex of the child		
Male	16,653	51.07
Female	15,957	48.93
Age of Mother		
15–19	1,570	4.81
20–24	6,752	20.71
25–29	9,589	29.41
30–34	6,600	20.24
35–39	5,039	15.45
40–44	2,218	6.80
45–49	842	2.58
Current marital status		
Never married	192	0.59
Married	29,311	89.88
Living together	849	2.60
Widowed	586	1.80
Divorced	1,089	3.34
Not living together	583	1.79
Partner’s education level		
No education	18,927	58.49
Primary	9,069	28.03
Secondary	3,266	10.09
Higher	882	2.73
Don’t know	215	0.66
Respondents educational		
No education	24,706	75.76
Primary	5,873	18.01
Secondary	1,719	5.27
Higher	312	0.96
Region		
Tigray	3,468	10.63
Affar	2,342	7.18
Amhara	4,404	13.51
Oromiya	5,897	18.08
Somali	2,374	7.28
Ben-Gumz	2,515	7.71
SNNP	4,933	15.13
Gambela	1,979	6.07
Harari	1,734	5.32
Addis Ababa	1,296	3.97
Dire Dawa	1,668	5.11
Place of residence		
Urban	5,080	15.58
Rural	27,530	84.42
Sex of household head		
Male	27,377	83.95
Female	5,233	16.05
Wealth indexQuintile		
Poorest	8,084	24.79
Poorer	5,989	18.37
Middle	6,207	19.03
Richer	5,817	17.84
Richest	6,513	19.97
Type of toilet facility		
Unimproved sanitation	27,053	84.87
Improved/modern sanitation	4,822	15.13
Source of drinking water		
Unimproved drinking water	17,324	54.36
Improved drinking water	14,546	45.64
Maternal Height		
≥ 145cm	617	2.26
<145cm	26,730	97.74
Respondent’s occupation		
Not working	17,301	53.29
working paid	6,441	19.84
Agricultural service	8,721	26.86
Partner’s occupation		
Not working	408	1.27
working paid	7,130	22.14
Agricultural service	24,673	76.60
Number of household members		
1–3 members	3,661	11.23
4–6 members	16,438	50.41
≥7 members	12,511	38.37
Number of under five children in the household		
≤2	27,523	84.40
>2	5,087	15.60

**Fig 1:**
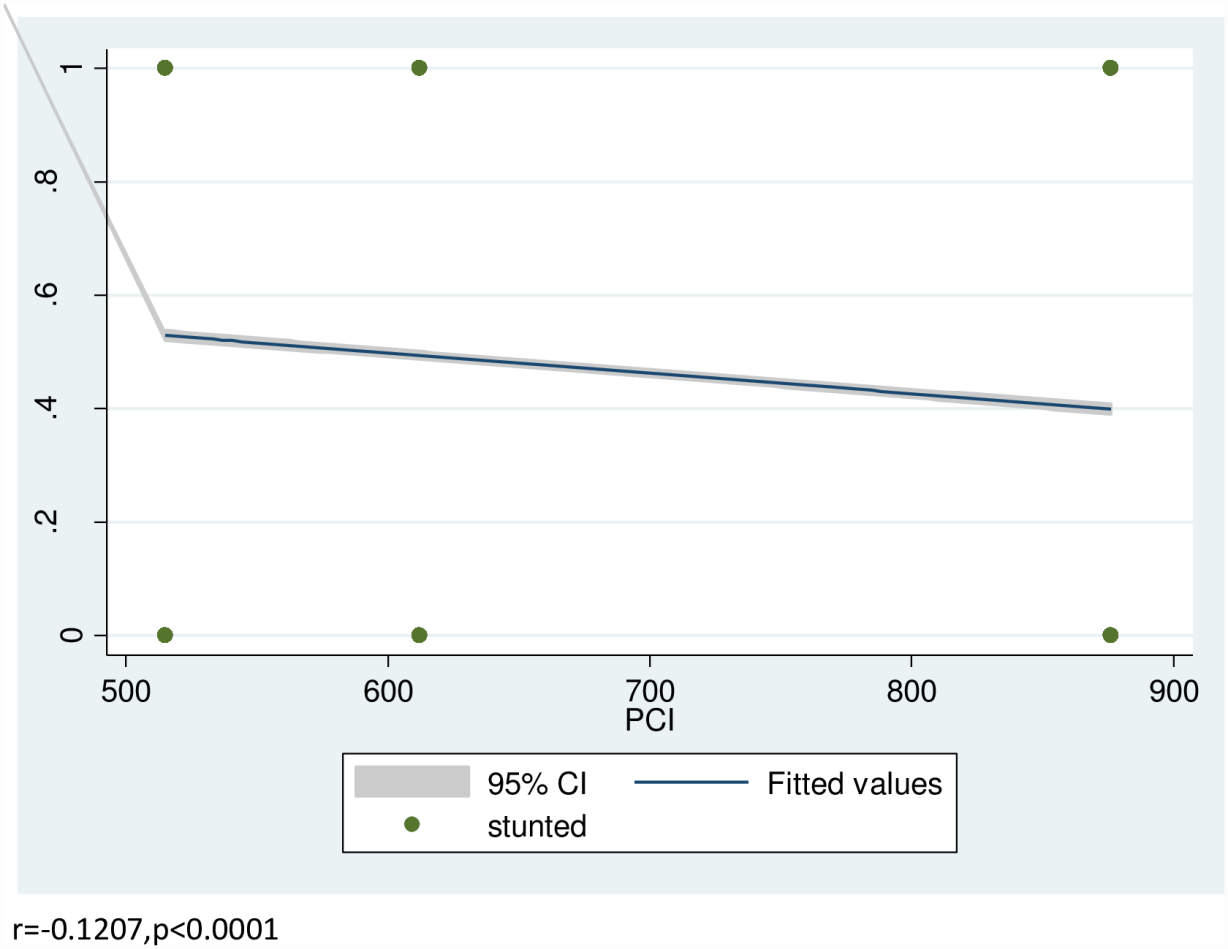
Correlation between prevalence of stunting and real per capita income (PCI).

**Fig 2:**
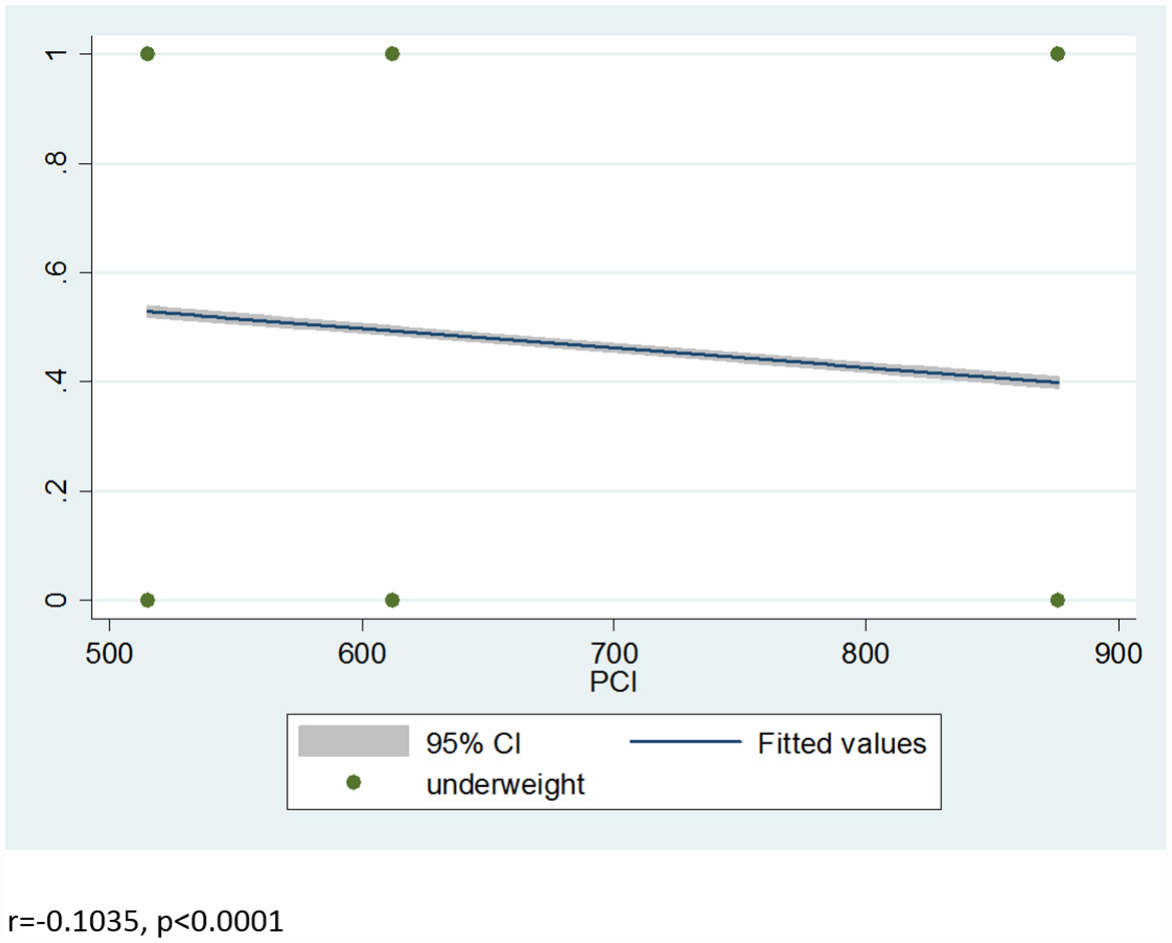
Correlation between prevalence of underweight and real per capita income (PCI).

**Fig 3:**
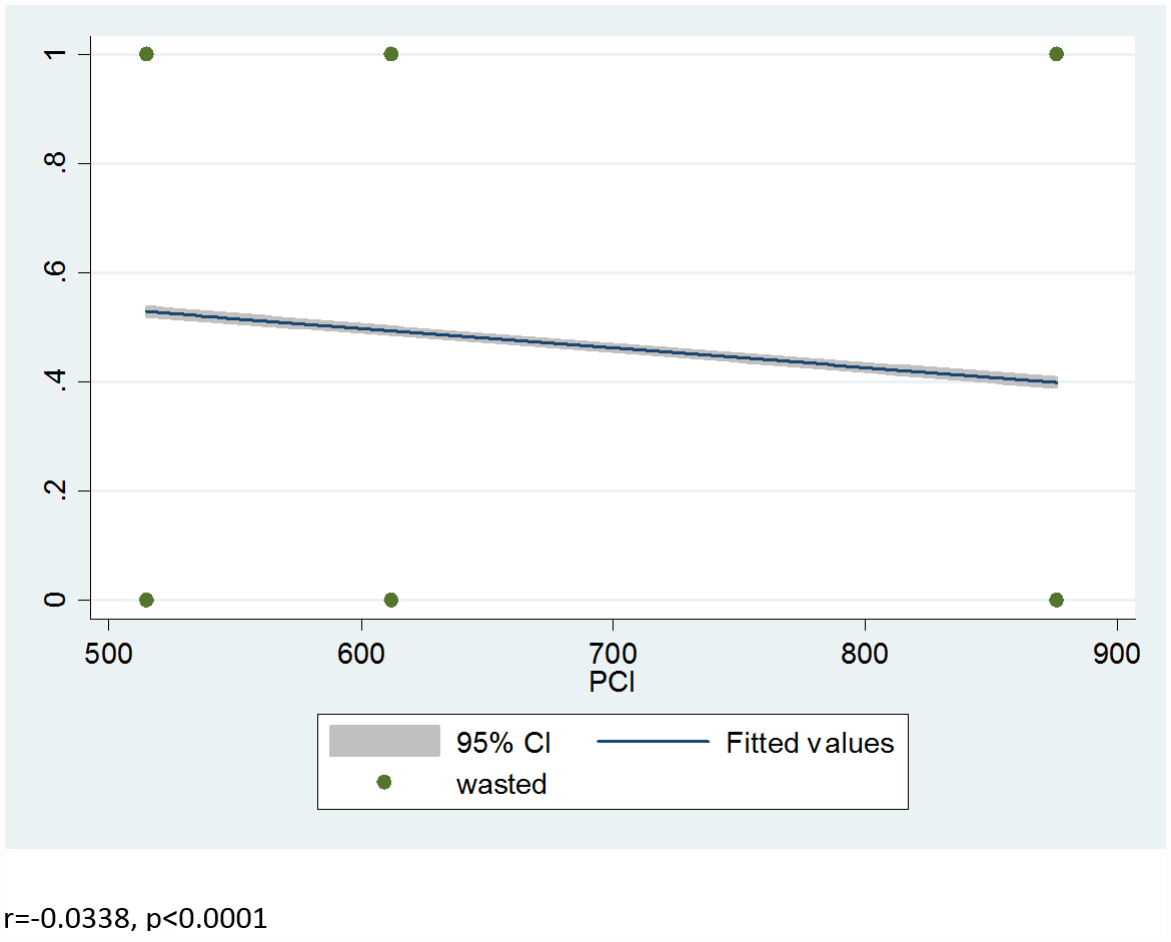
Correlation between prevalence of wasting and real per capita income (PCI).

From the total sample children, 11,296(46.7%) [46.0%-47.3%], 8,197(33.8%) [33.2%-34.4%] and 3,175(13.1%) [12.7%-13.5%] were stunted, underweight and wasted respectively. The proportions of stunting (r = -0.1207, p<0.0001), wasting (r = -0.0338, p<0.0001) and underweight (r = -0.1035, p<0.0001) were negatively correlated with the real per capita income (PCI) in Pearson correlation test [Figs 1–3]. The World Bank data shows that there was improvement in per-capita income of sample households in Ethiopia in the two sample periods by 18.85% and 43.14% from 2000 to 2005 and 2005 to 2010, respectively. Our models show that stunting [β = -0.002, SE = 0.0002, p<0.0001], underweight [β = -0.0014, SE = 0.0002, p<0.0001] and wasting [β = -0.0008, SE = 0.0002, p<0.0001] reduced with improvement in the PCI. Even after adjusted with child sex and age, economic growth reduce stunting [β = -0.002, SE = 0.0002, P<0.0001], underweight [β = -0.0015, SE = 0.0002, P<0.0001] and wasting [β = -0.0008, SE = 0.0002, p<0.0001] [S1–S3 Tables]. In the final model adjusted with all the covariates, economic growth substantially reduces stunting [β = -0.0016, SE = 0.00013, p<0.0001], underweight [β = -0.0014, SE = 0.0002, p<0.0001] and wasting [β = -0.0008, SE = 0.0002, p<0.0001] over a decade [Table 2].

**Table 2:** Final multilevel pooled regressions models that are predictor of early childhood undernutrtion among children age 6–59 months in Ethiopia.

Variables	Stunting			Underweight			Wasting		
	Coefficient	Stan.err	P-Value	Coefficient	Stan.err	P-Value	Coefficient	Stan.err	P-Value
PCI	-0.0016	0.00013	0.000	-0.0014	0.0002	0.000	-0.0008	0.0002	0.000
Current age of child									
0 years(ref)									
1 years	1.5375	0.0507	0.000	0.9129	0.0509	0.000	-0.0873	0.0569	0.125
2 years	1.8906	0.0511	0.000	1.0077	0.0504	0.000	-0.6561	0.0622	0.000
3 years	1.7545	0.0501	0.000	0.8411	0.0499	0.000	-1.0691	0.0670	0.000
4 years	1.5485	0.0506	0.000	0.8492	0.0507	0.000	-0.9144	0.0659	0.000
Sex									
Male (ref)									
Female	-0.1470	0.0295	0.000	-0.1395	0.0301	0.000	-0.2342	0.0409	0.000
Age of women									
15–19 (ref)									
20–24	0.0459	0.0849	0.589	0.0589	0.0875	0.501	0.0744	0.1089	0.494
25–29	-0.0089	0.0866	0.919	0.1137	0.0892	0.202	0.0328	0.1123	0.770
30–34	0.0064	0.0918	0.945	0.1927	0.0943	0.041	0.1515	0.1195	0.205
35–39	-0.0718	0.0953	0.451	0.1161	0.0978	0.235	0.1326	0.1246	0.287
40–44	-0.1297	0.1047	0.215	0.1251	0.1069	0.242	0.2660	0.1374	0.053
45–49	-0.3943	0.1276	0.002	-0.0989	0.1312	0.451	0.1321	0.1774	0.457
Region									
Tigray (ref)									
Affar	-0.0877	0.0957	0.360	0.1844	0.0967	0.056	0.5562	0.1181	0.000
Amhara	0.1496	0.0793	0.059	0.0928	0.0799	0.246	0.1031	0.1029	0.316
Oromiya	-0.2580	0.0768	0.001	-0.2652	0.0787	0.001	-0.0337	0.1017	0.741
Somali	-0.4384	0.0979	0.000	0.0440	0.0992	0.657	0.6660	0.1198	0.000
Ben-Gumz	-0.1071	0.0912	0.240	0.0720	0.0925	0.436	0.3360	0.1160	0.004
SNNP	0.0430	0.080	0.590	-0.0086	0.0810	0.915	0.0258	0.1053	0.807
Gambela	-0.6451	0.1007	0.000	-0.3404	0.1040	0.001	0.4389	0.1266	0.001
Harari	-0.5553	0.1066	0.000	-0.6298	0.1153	0.000	-0.2017	0.1491	0.176
Addis Ababa	-0.4840	0.1256	0.000	-0.8563	0.1534	0.000	-0.4147	0.2007	0.039
Dire Dawa	-0.5315	0.1081	0.000	-0.2018	0.1127	0.073	0.3572	0.1377	0.009
Place of residence									
Urban(ref)									
Rural	0.2769	0.0849	0.001	0.3798	0.0907	0.000	-0.0295	0.1164	0.800
Sex of household head									
Male (ref)									
Female	0.0653	0.0443	0.140	0.0571	0.0453	0.208	0.1262	0.0594	0.034
Wealth index Quintile									
Poorest(ref)									
Poorer	-0.0277	0.0471	0.556	-0.0226	0.0467	0.629	0.0303	0.0630	0.630
Middle	-0.0250	0.0485	0.607	-0.0123	0.0481	0.799	0.0879	0.0637	0.168
Richer	-0.0867	0.0514	0.092	-0.1950	0.0520	0.000	-0.1413	0.0710	0.047
Richest	-0.2693	0.0745	0.000	-0.3456	0.0774	0.000	-0.1748	0.1054	0.097
Type of toilet facility									
Unimproved sanitation(ref)									
Improved/modern sanitation	-0.1867	0.0557	0.001	-0.1750	0.0600	0.004	-0.1758	0.0824	0.033
Source of drinking water									
Unimproved drinking water(ref)									
Improved drinking water	-0.0193	0.0384	0.615	0.0195	0.0386	0.613	-0.0606	0.0509	0.233
Maternal Height									
≥ 145cm (ref)									
<145cm	-0.9254	0.1072	0.000	-0.6440	0.0982	0.000	0.0745	0.1425	0.601
Respondent’s occupation									
Not working(ref)									
Working paid	0.0384	0.1381	0.781	0.1241	0.1473	0.400	-0.5339	0.1642	0.001
Agricultural service	0.0172	0.1366	0.900	0.1138	0.1453	0.434	-0.3684	0.1609	0.022
Partner’s occupation									
Not working(ref)									
Working paid	0.0737	0.0424	0.082	0.0192	0.0446	0.668	-0.0133	0.0609	0.827
Agricultural service	0.0667	0.0420	0.112	0.0083	0.0424	0.846	0.0225	0.0569	0.693
Number of household members									
1–3 (ref)									
4–6	-0.0217	0.0582	0.709	-0.0706	0.0604	0.242	0.0321	0.0796	0.687
>7	-0.0192	0.0652	0.768	-0.1609	0.0675	0.017	-0.1026	0.0890	0.249
Number of under five children in the household									
≤2 (ref)									
<2	0.1180	0.0430	0.006	0.1945	0.0443	0.000	0.0904	0.0599	0.131
Partner’s education level									
No education(ref)									
Primary	-0.0589	0.0374	0.115	-0.1170	0.0382	0.002	-0.2232	0.0531	0.000
Secondary	-0.4333	0.0642	0.000	-0.3769	0.0695	0.000	-0.3111	0.0941	0.001
Higher	-0.7743	0.1147	0.000	-0.8666	0.1424	0.000	-0.2568	0.1639	0.117
Don’t know	0.3315	0.1819	0.068	0.0382	0.1836	0.835	-0.2146	0.2708	0.428
Constant	0.6058	0.2391	0.011	-0.1324	0.2451	0.589	-0.6425	0.3051	0.035
Random-effects									
Cluster Identity	0.1074	0.0783		0.0506	0.1807		0.0485	0.2886	
Year of interview	0.4606	0.0294		0.4818	0.0298		0.4983	0.0446	
LR test	0.0000			0.0000			0.0000		
Prob > *X*^2^	0.0000			0.0000			0.0000		

## Discussion

The study demonstrated that economic growth substantially reduced undernutrition in Ethiopia. The sampling process considered 83.95% male headed households, and more than 84.4% of the sample households were from rural parts of the country in which 76.6% of the total households were employed in agricultural activities. Nearly 49.0% of the sample children were female, which indicates that there was an approximate proportionality of sex ratio in the sampling process.

There is improvement in PCI of households in Ethiopia in the two sample period intervals by 18.85% and 43.14%, respectively. Given this, nearly 25% of the sample households were in the lowest quantile level of wealth index, and more than 52% of them had wealth index level below the middle quantile. In our study, the converted nutritional indices indicate that 46.7%, 33.8% and 13.1% of the children were stunted, underweight and wasted, respectively. Similarly, the pooled data of LMIC showed 35.6% of young children were stunted, 22.7% were underweight and12.8% were wasted [17].

The model result confirmed the hypothesis that economic development is associated with improved nutritional status via reducing underweight, wasted and stunting. Since majority of the sampled households were engaged in agriculture and the sector takes the significant share in the overall GDP, increment in agricultural productivity of the country in the last decades may play a vital role for nutrition improvement. This is in line with findings in other sub-Saharan African countries [2, 24] and calls for intervention in agricultural research and extension, infrastructure development and credit service provision [2, 4, 24, 25]. This might be due to increment in investment on health and other public infrastructures and associated with improvement in the availability and access to them. Improved PCI of household could enhance their expenditure for food and other basic health care services, which in turn might improve nutritional status of children [1, 4].

The micro-economic parameters of the household (wealth index and other variables) remain an important contributor to the reduction in stunting, underweight and wasting of children in Ethiopia. In addition our study showed that the reduction of the child malnutrition is more pronounced as we move in the higher/est wealth quintile (i.e. richest). Furthermore, we control for demographic, socio-economic and infrastructure related variables that might influence the outcome. We have evidence that, additional parameters like improved sanitation facility, accumulation of wealth and husband educational status of the respondents also play significant role for the reduction in child malnutrition [21]. Therefore, it is a holistic and comprehensive effort need to combat childhood malnutrition.

This contributes to empirical findings that confirm the association of household income and nutrition in sub-Saharan African countries [2]. Families in sub-Saharan Africa invest as much as 60% of their income for food related expenditures, and an improvement in wealth condition is often translated to better access to food and health care services. To facilitate further in undernutrition reduction in the country and achieving sustainable development goals(SDG), investments to boost agricultural production, stabilizing price volatilities that often harm the poor, and implementing policies targeted to increase income levels are crucial. As such, targeted agricultural programmes can complement nutrition and health investments by supporting livelihoods, enhancing access to diverse diets among the poor population. Evidence also showed that nutrition interventions had assured prominent effects on child health and development [26]. Despite the known challenge on expanding the quality and coverage of nutrition-specific interventions, targeted nutrition sensitive interventions with pro-poor development strategies can bring significant improvements [27]. So we argue that there is a need to focus on both nutrition-sensitive and nutrition-specific interventions that can boost child health and nutritional status [26]. Likewise, health and nutrition interventions, economic and social policies addressing poverty, trade, and agriculture should be seen as key elements in large scale country programs to have a great impact on reducing childhood undernutrition [28].

Contrary to the findings of this study, research in some other countries comes up with paradoxical findings on the effect of economic growth on child undernutrition. There are findings from some low income countries that showed that there is inverse association between economic growth and childhood underweight [20] and stunting, underweight, and wasting [21, 29]. According to Harttgen et.al (2012) study in Africa, there is a week associations between GDP per capita and underweight and stunting but in negative way and no association between GDP per capita and wasting [30]. Economic growth in various Indian states was not associated with a reduction in childhood stunting, underweight, and wasting [16] and no association was observed between average changes in the prevalence of child undernutrition outcomes and average growth of per-head GDP in a few low income countries [17]. The difference might be attributable to the analysis carried out among studies and methodological phenomena. Some use ecological analysis which doesn't account for individual-level factors and others use multilevel that can anticipate those variables as well as others factors apart from economic growth that may play crucial role in differencing the finding. There might be also a difference in domestic investments directions made while there is improvement in PCI.

This study had some limitations and should be interpreted with caution. Recall bias during interviewing of mothers, self-reported bias of respondents on children health status or respondents by themselves, measurement error might be present particularly on height and weight measurements of children, and lastly since the data are pooled cross-sectional in nature, it may be difficult to establish a cause and effect relationship. More importantly, the research does not incorporate program level interventions in terms of other national level sectoral program apart from health and nutrition like infrastructure development, water and sanitation (WASH).

In conclusion, economic growth has a significant effect on reducing child undernutrition problems in Ethiopia accompanied with socio-economic development and improvement. But a lot of factors play in the progressive reduction of childhood undernutrtion in the country in the past decades along with the economic growth. Other direct nutrition specific and nutrition sensitive interventions could also be recommended in order to have a greater impact on the massive reduction of childhood undernutrition and producing economically productive children that can end poverty and positively contribute to the economic development of Ethiopia.

## Supporting Information

S1 Table Multilevel pooled regressions models that are a potential predictor of stunting among children age 6–59 months in Ethiopia. (PDF)

S2 Table Multilevel pooled regressions models that are a potential predictor of underweight among children age 6–59 months in Ethiopia. (PDF)

S3 Table Multilevel pooled regressions models that are a potential predictor of wasting among children age 6–59 months in Ethiopia. (PDF)

## References

[1] Ranis G, Stewart F, Ramirez A: Economic Growth and Human Development *World development* 2000, 28(2):197–219

[2] Pauw K, Thurlow J: Agricultural growth, poverty, and nutrition in Tanzania. *Food Policy* 2011, 36:795–804.

[3] Tavneet Suri MA B, Gustav Ranis, Frances Stewart: Paths to Success: The Relationship Between Human Development and Economic Growth *World Development* 2011, 39(4):506–522.

[4] Ravallion M, Datt G: How important to India’s poor is the sectoral composition of economic growth?. *The World Bank Economic Review* 1996, 10(1):1–26.

[5] UN: Nutrition and the Post-2015: Sustainable Development Goals. A Technical Note. October 2014.

[6] Diao X, Hazell P, Thurlow J: The role of agriculture in African development *World Development* 2010, 38(10):1375–1383.

[7] Bhutta ZA A T, Black RE, Cousens S, Dewey K, Giugliani E, Haider BA, et al; Maternal and Child Undernutrition Study Group.: What works? Interventions for maternal and child undernutrition and survival. *Lancet* 2008, 371(9610):417–440 10.1016/S0140-6736(07)61693-6 18206226

[8] DNEAP: Poverty and Well Being in Mozambique: The Third National Assessment. National Directorate of Poverty Analysis and Studies, Ministry of Planning and Development, Maputo, Mozambique. 2010.

[9] Bank W: World Development Indicators Online Database. World Bank, Washington, DC, USA 2010.

[10] Maye´n Ana-Lucia M-V P, Paccaud Fred, Bovet Pascal, and Stringhini Silvia Socioeconomic determinants of dietary patterns in low- and middle-income countries: a systematic review *Am J Clin Nutr* 2014, 100(6):1520–1531 10.3945/ajcn.114.089029 25411287

[11] O'Donnella O, Nicolásb ÁL, Doorslaerc EV: Growing richer and taller: Explaining change in the distribution of child nutritional status during Vietnam's economic boom. *Journal of Development Economics* 2009, 88(1):45–58.

[12] Bhutta ZA D J, Rizvi A, Gaffey MF, Walker N, Horton S, Webb P, Lartey A, Black RE; Lancet Nutrition Interventions Review Group; Maternal and Child Nutrition Study Group.: Evidence-based interventions for improvement of maternal and child nutrition: what can be done and at what cost? *Lancet* 2013, 382(9890):452–477 10.1016/S0140-6736(13)60996-4 23746776

[13] FMOH: Program Implementation Manual of National Nutrition Program (NNP)—I (July 2008 –June 2013). Federal Ministry of Health [Ethiopia] Addis Ababa,Ethiopia 2008.

[14] FMoH/UNICEF/EU: Situation Analysis of the Nutrition Sector in Ethiopia: 2000–2015. Ethiopian Federal Ministry of Health, UNICEF and European Commission Delegation. Addis Ababa, Ethiopia 2016.

[15] Alderman H, Garcia M: Food Security and Health Security: Explaining the Levels of Nutritional Status in Pakistan. *Economic Development and Cultural Change* 1994, 42 (3):485–508

[16] Subramanyam M, Kawachi I, Berkman L, Subramanian S: Is economic growth associated with reduction in child undernutrition in India?. *PLoS Med* 2011 8(3):e1000424 10.1371/journal.pmed.1000424 21408084 PMC3050933

[17] Vollmer S, Harttgen K, Subramanyam M, Finlay J, Klasen S, Subramanian S: Association between economic growth and early childhood undernutrition: evidence from 121 Demographic and Health Surveys from 36 low-income and middle-income countries *Lancet Glob Health* 2014, 2(4):e225–234 10.1016/S2214-109X(14)70025-7 25103063

[18] Alderman H, Appleton S, Haddad L, Song L, Yohannes Y: Reducing Child Malnutrition: How Far Does Income Growth Take Us? *Centre for Research in Economic Development and International Trade(CREDIT) Research paper* 2001, No. 01/05.

[19] Edmonds E: How well do improvements in economic status track nonmonetary measures of well-being? Evidence from child height. Dartmouth College, Hannover, NH 2004.

[20] Smith L, Haddad L: How potent is economic growth in reducing undernutrition? What are the pathways of impact? New cross-country evidence *Econ Dev Cult Change* 2002 51 55–76

[21] Harttgen K, Klasen S, Vollmer S: Economic Growth and Child Undernutrition in sub-Saharan Africa. *Population and Development Review* 2013, 39(3):397–412.

[22] Bank W: World Development Indicators. The World Bank (Available at http://data.worldbank.org/country/ethiopia, accessed at Febuaruy 20, 2016). 2012.

[23] WHO/UNICEF: Progress on Drinking Water and Sanitation: 2012 Update. New York: World Health Organization and United Nations Children's Fund Joint Monitoring Programme (JMP) for Water Supply and Sanitation: Available at http://www.wssinfo.org/fileadmin/user_upload/resources/JMP_report_2014_webEng.pdf. 2014.

[24] Thirtle C, Lin L, Piesse J: The impact of research-led agricultural productivity growth on poverty reduction in Africa, Asia and Latin America *World Development* 2003, 31(12):1959–1975

[25] Sanchez P, Denning G, Nziguheba G: The African green revolution moves forward *Food Security* 2009, 1:37–44

[26] Ruel M, Alderman H, Group MaCNS: Nutrition-sensitive interventions and programmes: how can they help to accelerate progress in improving maternal and child nutrition?. *Lancet* 2013, 382(9891). 10.1016/S0140-6736(13)60843-0 23746780

[27] Gillespie S, Haddad L, Mannar V, Menon P, N NN, Group MaCNS: The politics of reducing malnutrition: building commitment and accelerating progress *Lancet* 2013, 382(9891). 10.1016/S0140-6736(13)60842-9 23746781

[28] Bryce J, Coitinho D, Darnton-Hill I, Pelletier D, Pinstrup-Andersen P, Group MaCUS: Maternal and child undernutrition: effective action at national level *Lancet* 2008, 371(9611). 10.1016/S0140-6736(07)61694-8 18206224

[29] Kenneth H, Stephan K, Sebastian V: Economic Growth and Child Undernutrition in sub-Saharan Africa. *Population and Development Review* 2013, 39(3):397–412.

[30] Harttgen K, Klasen S, Vollmer S: Economic Growth and Child Undernutrition in Africa. United Nations Development program (UNDP) Working Paper (WP) 2012–013: February 2012.

