# Supplementary material for: Does Economic Growth Reduce Childhood Undernutrition in Ethiopia?
Source: PLoS One. 2016 Aug 10;11(8):e0160050. doi: 10.1371/journal.pone.0160050 (PMC4979960; doi:10.1371/journal.pone.0160050)
Supplement: S1 Table — (PDF) [file pone.0160050.s001.pdf]

S1 Table. Multilevel pooled regressions models that are a potential predictor of stunting among children age 6-59 months in Ethiopia

Variables		Model_1			Model_2			Model_3		
		Coefficient	Stan.err	P-Value	Coefficient	Stan.err	P-Value	Coefficient	Stan.err	P-Value
PCI		-0.002	0.0002	0.000	-0.002	0.0002	0.000	-0.0016	0.00013	0.000
Current age of child	0 years(ref)									
	1 years	--	---	--	1.4923	0.0491	0.000	1.5375	0.0507	0.000
	2 years				1.8454	0.0493	0.000	1.8906	0.0511	0.000
	3 years				1.7378	0.0482	0.000	1.7545	0.0501	0.000
	4 years				1.5203	0.0486	0.000	1.5485	0.0506	0.000
Sex	Male (ref)									
	Female				-0.154	0.0288	0.000	-0.1470	0.0295	0.000
Age of women	15-19 (ref)									
	20-24							0.0459	0.0849	0.589
	25-29							-0.0089	0.0866	0.919
	30-34							0.0064	0.0918	0.945
	35-39							-0.0718	0.0953	0.451
	40-44							-0.1297	0.1047	0.215
	45-49							-0.3943	0.1276	0.002
Region	Tigray (ref)									
	Affar							-0.0877	0.0957	0.360
	Amhara							0.1496	0.0793	0.059
	Oromiya							-0.2580	0.0768	0.001
	Somali							-0.4384	0.0979	0.000
	Ben-Gumz							-0.1071	0.0912	0.240
	SNNP							0.0430	0.080	0.590
	Gambela							-0.6451	0.1007	0.000
	Harari							-0.5553	0.1066	0.000
	Addis Ababa							-0.4840	0.1256	0.000
	Dire Dawa							-0.5315	0.1081	0.000
Place of residence	Urban(ref)									
	Rural							0.2769	0.0849	0.001

Sex of household head	Male (ref)									
	Female							0.0653	0.0443	0.140
Wealth index Quintile	Poorest(ref)									
	Poorer							-0.0277	0.0471	0.556
	Middle							-0.0250	0.0485	0.607
	Richer							-0.0867	0.0514	0.092
	Richest							-0.2693	0.0745	0.000
Type of toilet facility	unimproved sanitation									
	Improved/modern sanitation							-0.1867	0.0557	0.001
Source of drinking water	unimproved drinking water									
	improved drinking water							-0.0193	0.0384	0.615
Maternal Height	≥ 145									
	<145cm							-0.9254	0.1072	0.000
Respondent’s occupation	Not working									
	working paid							0.0384	0.1381	0.781
	Agricultural service							0.0172	0.1366	0.900
Partner’s occupation	Not working(ref)									
	working paid							0.0737	0.0424	0.082
	Agricultural service							0.0667	0.0420	0.112
Number of household members	1-3 (ref)									
	4-6							-0.0217	0.0582	0.709
	>7							-0.0192	0.0652	0.768
Number of under five children in the household	≤2									
	<2							0.1180	0.0430	0.006

Partner's education level	No education(ref)									
	Primary							-0.0589	0.0374	0.115
	Secondary							-0.4333	0.0642	0.000
	Higher							-0.7743	0.1147	0.000
	Don't know							0.3315	0.1819	0.068
Constant		0.8896	0.0897	0.000	-0.307	0.1028	0.003	0.6058	0.2391	0.011
Random-effects										
Cluster Identity		0.1191	0.0802		0.1236	0.0897		0.1074	0.0783	
Year of interview		0.5876	0.0276		0.6494	0.0292		0.4606	0.0294	
LR test		0.0000			0.0000			0.0000		
Prob > $\chi 2$		0.0000			0.0000			0.0000		
