# Supplementary material for: Does Economic Growth Reduce Childhood Undernutrition in Ethiopia?
Source: PLoS One. 2016 Aug 10;11(8):e0160050. doi: 10.1371/journal.pone.0160050 (PMC4979960; doi:10.1371/journal.pone.0160050)
Supplement: S2 Table — (PDF) [file pone.0160050.s002.pdf]

S2 Table. Multilevel pooled regressions models that are a potential predictor of underweight among children age 6-59 months in Ethiopia

Variables		Model_1			Model_2			Model_3		
		Coefficient	Stan.err	P-Value	Coefficient	Stan.err	P-Value	Coefficient	Stan.err	P-Value
PCI		-0.0014	0.0002	0.000	-0.0015	0.0002	0.000	-0.0014	0.0002	0.000
Current age of child	0 years(ref)									
	1 years				0.8786	0.0493	0.000	0.9129	0.0509	0.000
	2 years				0.9941	0.0487	0.000	1.0077	0.0504	0.000
	3 years				0.8585	0.0481	0.000	0.8411	0.0499	0.000
	4 years				0.8650	0.0487	0.000	0.8492	0.0507	0.000
Sex	Male (ref)									
	Female				-0.1361	0.0293	0.000	-0.1395	0.0301	0.000
Age of women	15-19 (ref)									
	20-24							0.0589	0.0875	0.501
	25-29							0.1137	0.0892	0.202
	30-34							0.1927	0.0943	0.041
	35-39							0.1161	0.0978	0.235
	40-44							0.1251	0.1069	0.242
	45-49							-0.0989	0.1312	0.451
Region	Tigray (ref)									
	Affar							0.1844	0.0967	0.056
	Amhara							0.0928	0.0799	0.246
	Oromiya							-0.2652	0.0787	0.001
	Somali							0.0440	0.0992	0.657
	Ben-Gumz							0.0720	0.0925	0.436
	SNNP							-0.0086	0.0810	0.915
	Gambela							-0.3404	0.1040	0.001
	Harari							-0.6298	0.1153	0.000
	Addis Ababa							-0.8563	0.1534	0.000

	Dire Dawa							-0.2018	0.1127	0.073
Place of residence	Urban(ref)									
	Rural							0.3798	0.0907	0.000
Sex of household head	Male (ref)									
	Female							0.0571	0.0453	0.208
Wealth index Quintile	Poorest(ref)									
	Poorer							-0.0226	0.0467	0.629
	Middle							-0.0123	0.0481	0.799
	Richer							-0.1950	0.0520	0.000
	Richest							-0.3456	0.0774	0.000
Type of toilet facility	unimproved sanitation									
	Improved/modern sanitation							-0.1750	0.0600	0.004
Source of drinking water	unimproved drinking water									
	improved drinking water							0.0195	0.0386	0.613
Maternal Height	≥ 145cm (ref)									
	<145cm							-0.6440	0.0982	0.000
Respondent's occupation	Not working(ref)									
	working paid							0.1241	0.1473	0.400
	Agricultural service							0.1138	0.1453	0.434
Partner's occupation	Not working(ref)									
	working paid							0.0192	0.0446	0.668
	Agricultural service							0.0083	0.0424	0.846
Number of household members	1-3 (ref)									
	4-6							-0.0706	0.0604	0.242

	>7							-0.1609	0.0675	0.017
Number of under five children in the household	≤2 (ref)									
	<2							0.1945	0.0443	0.000
Partner's education level	No education(ref)									
	Primary							-0.1170	0.0382	0.002
	Secondary							-0.3769	0.0695	0.000
	Higher							-0.8666	0.1424	0.000
	Don't know							0.0382	0.1836	0.835
Constant		0.1520	0.0966	0.116	-0.4946	0.1048	0.000	-0.1324	0.2451	0.589
Random-effects										
Cluster Identity		0.00003	0.1166		0.0001	0.1283		0.0506	0.1807	
Year of interview		0.6443	0.0239		0.6616	0.0242		0.4818	0.0298	
LR test		0.0000			0.0000			0.0000		
Prob > $\chi2$		0.0000			0.0000			0.0000		
