# Supplementary material for: Does Economic Growth Reduce Childhood Undernutrition in Ethiopia?
Source: PLoS One. 2016 Aug 10;11(8):e0160050. doi: 10.1371/journal.pone.0160050 (PMC4979960; doi:10.1371/journal.pone.0160050)
Supplement: S3 Table — (PDF) [file pone.0160050.s003.pdf]

S3 Table. Multilevel pooled regressions models that are a potential predictor of wasting among children age 6-59 months in Ethiopia

Variables		Model_1			Model_2			Model_3		
		Coefficient	Stan.err	P-Value	Coefficient	Stan.err	P-Value	Coefficient	Stan.err	P-Value
PCI		-0.0008	0.0002	0.000	-0.0008	0.0002	0.000	-0.0008	0.0002	0.000
Current age of child	0 years(ref)									
	1 years				-0.0863	0.0553	0.119	-0.0873	0.0569	0.125
	2 years				-0.6315	0.0603	0.000	-0.6561	0.0622	0.000
	3 years				-1.0389	0.0651	0.000	-1.0691	0.0670	0.000
	4 years				-0.8692	0.0635	0.000	-0.9144	0.0659	0.000
Sex	Male (ref)									
	Female				-0.2176	0.0401	0.000	-0.2342	0.0409	0.000
Age of women	15-19 (ref)									
	20-24							0.0744	0.1089	0.494
	25-29							0.0328	0.1123	0.770
	30-34							0.1515	0.1195	0.205
	35-39							0.1326	0.1246	0.287
	40-44							0.2660	0.1374	0.053
	45-49							0.1321	0.1774	0.457
Region	Tigray (ref)									
	Affar							0.5562	0.1181	0.000
	Amhara							0.1031	0.1029	0.316
	Oromiya							-0.0337	0.1017	0.741
	Somali							0.6660	0.1198	0.000
	Ben-Gumz							0.3360	0.1160	0.004
	SNNP							0.0258	0.1053	0.807
	Gambela							0.4389	0.1266	0.001
	Harari							-0.2017	0.1491	0.176
	Addis Ababa							-0.4147	0.2007	0.039
	Dire Dawa							0.3572	0.1377	0.009
Place of residence	Urban(ref)									
	Rural							-0.0295	0.1164	0.800

Sex of household head	Male (ref)									
	Female							0.1262	0.0594	0.034
Wealth index Quintile	Poorest(ref)									
	Poorer							0.0303	0.0630	0.630
	Middle							0.0879	0.0637	0.168
	Richer							-0.1413	0.0710	0.047
	Richest							-0.1748	0.1054	0.097
Type of toilet facility	unimproved sanitation(ref)									
	Improved/modern sanitation							-0.1758	0.0824	0.033
Source of drinking water	unimproved drinking water(ref)									
	improved drinking water							-0.0606	0.0509	0.233
Maternal Height	≥ 145cm (ref)									
	<145cm							0.0745	0.1425	0.601
Respondent's occupation	Not working(ref)									
	working paid							-0.5339	0.1642	0.001
	Agricultural service							-0.3684	0.1609	0.022
Partner's occupation	Not working(ref)									
	working paid							-0.0133	0.0609	0.827
	Agricultural service							0.0225	0.0569	0.693
Number of household members	1-3 (ref)									
	4-6							0.0321	0.0796	0.687
	>7							-0.1026	0.0890	0.249
Number of under five children in the household	≤2 (ref)									
	<2							0.0904	0.0599	0.131

Partner's education level	No education(ref)									
	Primary							-0.2232	0.0531	0.000
	Secondary							-0.3111	0.0941	0.001
	Higher							-0.2568	0.1639	0.117
	Don't know							-0.2146	0.2708	0.428
Constant		-1.5287	0.1097	0.000	-0.9630	0.1188	0.000	-0.6425	0.3051	0.035
Random-effects										
Cluster Identity		0.0001	0.3196		0.0001	0.3319		0.0485	0.2886	
Year of interview		0.5926	0.0316		0.6193	0.0322		0.4983	0.0446	
LR test		0.0000			0.0000			0.0000		
Prob > $\chi 2$		0.0000			0.0000			0.0000		
